# Novel *N*-Acyl Homoserine Lactone-Degrading Bacteria Isolated From Penicillin-Contaminated Environments and Their Quorum-Quenching Activities

**DOI:** 10.3389/fmicb.2019.00455

**Published:** 2019-03-14

**Authors:** Hiroyuki Kusada, Yu Zhang, Hideyuki Tamaki, Nobutada Kimura, Yoichi Kamagata

**Affiliations:** ^1^Bioproduction Research Institute, National Institute of Advanced Industrial Science and Technology, Tsukuba, Japan; ^2^State Key Laboratory of Environmental Aquatic Chemistry, Research Center for Eco-Environmental Sciences, University of Chinese Academy of Sciences, Chinese Academy of Sciences, Beijing, China; ^3^JST ERATO Nomura Microbial Community Control Project, University of Tsukuba, Tsukuba, Japan

**Keywords:** β-lactam antibiotic resistance, quorum sensing, quorum quenching, AHL-acylase, AHL-lactonase

## Abstract

*N*-Acyl homoserine lactones (AHLs) are signaling molecules used in the quorum sensing (QS) of Gram-negative bacteria. Some bacteria interfere with the QS system using AHL-inactivating enzymes, commonly known as quorum-quenching (QQ) enzymes. We have recently isolated a new QQ bacterium showing high resistance to multiple β-lactam antibiotics, and its QQ enzyme (MacQ) confers β-lactam antibiotic resistance and exhibits QQ activities. This observation suggests the possibility of isolating novel QQ bacteria from β-lactam antibiotic-resistant bacteria. In this direction, we attempted to isolate penicillin G (PENG)-resistant bacteria from penicillin-contaminated river sediments and activated sludge treating penicillin-containing wastewater and characterize their QQ activities. Of 19 PENG-resistant isolates, six isolates showed high QQ activity toward a broad range of AHLs, including AHLs with 3-oxo substituents. Five of the six AHL-degraders showed AHL-acylase activity and hydrolyzed the amide bond of AHLs, whereas the remaining one strain did not show AHL-acylase activity, suggesting that this isolate may likely possess alternative degradation mechanism such as AHL-lactonase activity hydrolyzing the lactone ring of AHLs. The 16S rRNA gene sequence analysis results categorized these six AHL-degrading isolates into at least five genera, namely, *Sphingomonas* (*Alphaproteobacteria*), *Diaphorobacter* (*Betaproteobacteria*), *Acidovorax* (*Betaproteobacteria*), *Stenotrophomonas* (*Gammaproteobacteria*), and *Mycobacterium* (*Actinobacteria*); of these, *Mycobacterium* sp. M1 has never been known as QQ bacteria. Moreover, multiple β-lactam antibiotics showed high minimum inhibitory concentrations (MICs) when tested against all of isolates. These results strongly demonstrate that a wide variety of β-lactam antibiotic-resistant bacteria possess QQ activities. Although the genetic and enzymatic elements are yet unclear, this study may infer the functional and evolutionary correlation between β-lactam antibiotic resistance and QQ activities.

## Introduction

Bacteria communicate with one another using chemical signaling molecules. The sensing of auto-inducers allows bacteria to distinguish between low and high cell population densities as well as to adjust the gene expression in response to changes in cell number. This process, termed as quorum sensing (QS), allows bacterial cells to coordinately control the gene expression in the community. *N*-Acyl homoserine lactone (AHL)-dependent QS has been known to regulate many bacterial behaviors such as virulence (Givskov et al., 1998; Lindum et al., 1998; Burr et al., 2006; Xu et al., 2006) and biofilm formation (Hammer and Bassler, 2003; Labbate et al., 2004).

Over the past decade, several studies have been directed to understand the phenomenon of quorum quenching (QQ), a signal interference process that attenuates QS systems. QQ is a typical characteristic of a variety of organisms that degrade AHLs by enzymatic reactions (Dong and Zhang, 2005). Microbial communities harbor counter constituents and exhibit mechanisms as one of their survival strategies that hinder or compete with QS bacteria. Phylogenetically diverse AHL-inactivating bacteria that belong to the phyla *Proteobacteria* (genera *Acidovorax, Acinetobacter, Achromobacter, Alcaligenes, Alteromonas, Agrobacterium, Bosea, Brevundimonas, Comamonas, Delftia, Diaphorobacter, Klebsiella, Mesorhizobium, Ochrobactrum, Pseudomonas, Ralstonia, Roseomonas, Shewanella, Sphingomonas, Stenotrophomonas*, and *Variovorax*), *Bacteroidetes* (*Chryseobacterium, Flaviramulus*, and *Tenacibaculum*), and *Cyanobacteria* (*Nostoc*) have been isolated and characterized as QQ bacteria (Leadbetter and Greenberg, 2000; Flagan et al., 2003; Hu et al., 2003; Huang et al., 2003; Lin et al., 2003; Park et al., 2003; Uroz et al., 2003; Sio et al., 2006; Yoon et al., 2006; Romero et al., 2008, 2010; Chan et al., 2011; Christiaen et al., 2011; Mahmoudi et al., 2011; Chen et al., 2012; Wang et al., 2012; Zhang et al., 2013; Torres et al., 2016; Kusada et al., 2017). Furthermore, Gram-positive bacteria within the phyla, *Actinobacteria* (*Arthrobacter, Microbacterium, Nocardioides, Rhodococcus, Staphylococcus*, and *Streptomyces*), *Deinococcus-Thermus* (*Deinococcus*), and *Firmicutes* (*Bacillus* and *Solibacillus*) have been found to exhibit QQ activities, indicating that phylogenetically diverse bacteria may quench the AHL-based QS (Dong et al., 2000; Lee et al., 2002; Park et al., 2003; Uroz et al., 2003; d’Angelo-Picard et al., 2005; Wang et al., 2010; Morohoshi et al., 2012; Koch et al., 2014; Chan et al., 2015).

**FIGURE 1:**
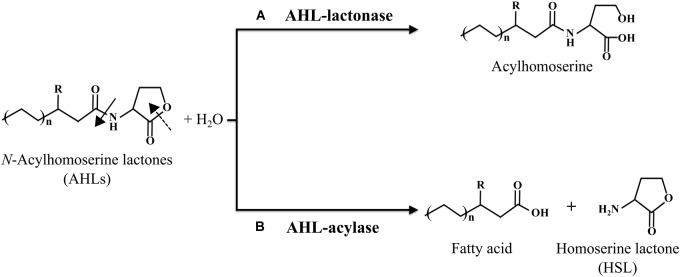
The general structure of AHL signals and the corresponding degradation mechanisms of AHL-lactonase **(A)** and AHL-acylase **(B)**. Cleavage of the lactone ring by an AHL-lactonase enzyme (dashed arrow) yields the corresponding acyl homoserine. Cleavage of the amide bond by an AHL-acylase enzyme (filled arrow) yields the corresponding fatty acid and homoserine lactone (HSL) ring.

To date, two different types of QQ enzymes have been identified, namely, AHL-lactonase and AHL-acylase. In the earliest study, AHL-lactonase gene (*aiiA*) from *Bacillus* species strain 240B1 was cloned and shown to encode the lactonase enzyme that hydrolyzes the ester bond of the lactone ring to produce acyl homoserine (Figure 1A) (Dong et al., 2000). On the other hand, *Variovorax paradoxus* strain was found to degrade AHLs by an acylase, wherein the amide bond between the homoserine lactone (HSL) ring and the acyl chain was cleaved to release HSL and fatty acid (Figure 1B) (Leadbetter and Greenberg, 2000). Database retrieval by Kalia et al. (2011) for homologs of the characterized AHL-lactonases and -acylases in complete bacterial genomes have shown that the relatives of these enzymes are widespread in a diverse array of organisms, suggestive of the ubiquity of QQ systems in natural microbial communities (Kalia et al., 2011).

We have recently isolated a novel AHL-degrading bacterium, *Acidovorax* sp. MR-S7, that exhibits high resistance to multiple β-lactam antibiotics (Kusada et al., 2017). Our study revealed a novel AHL-acylase (MacQ) from MR-S7 that confers β-lactam antibiotic resistance and exhibits QQ activity (Kusada et al., 2017). Therefore, we hypothesize that functionally novel and hitherto-unidentified QQ bacteria may be present among multiple β-lactam antibiotic resistant bacteria. However, very little is known about organisms that exhibit both QQ activities and β-lactam antibiotic resistance. Such microorganisms are very rare, and only two strains, *Pseudomonas aeruginosa* PAO1 and *Acidovorax* sp. MR-S7, have been reported so far. Here, we report the characterization of the QQ activity and antibiotic resistance of multiple newly isolated β-lactam antibiotic-resistant bacteria and discuss their phylogenetic relevance with other known QQ bacteria.

## Materials and Methods

### Sample Description

The environmental samples were obtained from the wastewater treatment plants in the penicillin G (PENG) production facility of the North China Pharmaceutical Group Corporation (NCPGC) and the receiving river, Wangyang River in Hebei Province, China. The wastewater from the PENG production factory is discharged into the Wangyang River after biological treatment (activated sludge treatment system), including anaerobic treatment, hydrolyzation and acidification, primary aerobic treatment, and secondary aerobic treatment. The average hydraulic residence time for each unit is about 30 h. The annual output of the excess sludge from the wastewater treatment plant (WWTP), which has been in operation since the 1990s, is 1,200 tons (dry weight). The wastewater in this plant contains 80% of wastewater from PENG production and 20% wastewater from the production of other antibiotics (cefalexin [CEFL], cefadroxil [CEFD], ampicillin [AMP], and amoxicillin [AMO]). The detected concentrations of PENG for activated sludge, river water, and sediment samples were 0.076 mg/kg, 0.000031 mg/L, and no detection, respectively. These low concentrations of PENG after the treatment clearly indicate the wastewater from PENG production could be properly treated by the activated sludge treatment system, suggesting that PENG-degrading bacteria would be present in the activated sludge sample.

The activated sludge and river sediment samples were obtained from the sludge concentration tanks that collect sludge samples from all of the biological treatment reactors and Wangyang River near the WWTP discharge outlet, respectively. The samples were collected in brown glass bottles that had been successively washed with tap water, ultra-pure water, and hexane and stored at 4°C in the dark.

### Isolation of Antibiotic-Resistant Bacteria Using Gellan Gum Medium

In this study, gellan gum-based media were used for the cultivation and isolation of antibiotic-resistant and AHL-degrading bacteria from the environmental samples, as gellan was found to be more effective than agar for the culturing of a diverse array of uncultured microorganisms (Tamaki et al., 2005, 2009). Activated sludge and sediment samples (500 μL) were suspended in sterile water and subjected to 10-fold serial dilutions. A series of medium plates (R2A-gellan gum) supplemented with 15, 30, 50, and 100 μg/mL of PENG were inoculated with 100-μL aliquots from different dilutions and incubated at 20°C for 4 weeks in the dark under aerobic conditions. Individual PENG-resistant colonies were purified thrice using fresh medium plates supplemented with 100 μg/mL of PENG and stored in 20% glycerol at -80°C.

The composition of R2A was as follows (per liter): 0.5 g each of yeast extract, peptone, acid hydrolysate of casein, glucose, and soluble starch; 0.3 g each of dipotassium phosphate and sodium pyruvate; and 0.05 g of magnesium sulfate. The pH values of these media were adjusted to 7.0 with 10 mM potassium phosphate buffer. The media were solidified with gellan gum (Wako, Tokyo, Japan) at a final concentration of 1.0%.

### Identification and Phylogenic Analysis of Antibiotic-Resistant Isolates and AHL-Degrading Bacterial Strains

Phylogenetic identification of the antibiotic-resistant isolates was performed using the 16S rRNA gene sequencing analysis. DNA templates for polymerase chain reaction (PCR) amplification from isolates were extracted by FastDNA^®^ Spin Kit (MP Biomedicals, Illkirch, France). The 16S rRNA genes of the isolates were PCR-amplified from the colonies using the primers 27F (5^′^-AGATTTGATCCTGGCTCAG-3^′^) and 1492R (5^′^-GGTTACCTTGTTACGACTT-3^′^). The PCR conditions included denaturation at 95°C for 9 min, followed by 40 cycles at 95°C for 1 min, 50°C for 1 min, and 72°C for 2 min. The final extension was performed at 72°C for 10 min. PCR products were purified with a MicroSpin S-400 HR column and used as templates for sequencing. Sequencing was performed with the primer 907R (5^′^-CCGTCAATTCMTTTGAGTTT-3^′^), a DTCS-Quick Start kit (Beckman Coulter, Fullerton, CA, United States), and a CEQ-2000 automated sequence analyzer (Beckman). The sequences of the resistant bacterial 16S rRNA gene clones with a range of about 500–600 bases were determined. All the 16S rRNA gene sequences of the antibiotic-resistant isolates were compared with those in the GenBank database ^1^ using the BLAST program (Altschul et al., 1990). For AHL-degrading bacteria, almost full 16S rRNA gene sequences (approximately 1,500 bp) were determined using primers 27F, 530F (5^′^-GTGCCAGCMGCCGCGG-3^′^), 907R, 1100F (5^′^-AAGTCCCGCAACGAGCGCA-3^′^), and 1492R. Multiple alignments of the 16S rRNA gene sequences of PENG-resistant isolates capable of degrading AHLs were performed with previously known QQ bacteria. The phylogenetic tree was constructed by neighbor-joining method using MEGA software (Tamura et al., 2011). Bootstrap values were estimated using neighbor-joining and maximum-likelihood methods (each 1,000 replications).

### Bioassay of AHL-Degrading Activity

The tested AHL compounds included non-substituted C_6_-HSL, C_8_-HSL, C_10_-HSL, C_12_-HSL, and C_14_-HSL as well as substituted 3-oxo-C_6_-HSL, 3-oxo-C_8_-HSL, 3-oxo-C_10_-HSL, 3-oxo-C_12_-HSL, and 3-oxo-C_14_-HSL. AHL degradation assay was performed using AHL-detectable reporter (biosensor) strains, *Escherichia coli* JB525-MT102 (pJBA132) and *P. putida* F117 (pKR-C12) (Andersen et al., 2001; Steidle et al., 2001). Briefly, exogenous permeable AHL molecules bind to a LuxR-type response regulator protein and constitute AHL-LuxR complex within biosensor strains. The AHL-LuxR complex binds to the promoter region of a GFP reporter gene. Therefore, the degradation of AHLs by the sample could be characterized with a decrease or extinction in GFP fluorescence.

For the whole-cell assay to determine the AHL-degrading ability, 3-day cultures of the isolates were washed and re-suspended in 100 mM potassium phosphate buffer (pH 6.5). A total volume of 50 μL of the cell re-suspension and an equal volume of AHL (final concentration, 20 μM) were mixed and the mixture was incubated at 30°C in the dark with gentle agitation. The samples were treated with ultraviolet irradiation for 1 h to stop the reaction, and the reaction mixtures were diluted to an appropriate concentration and loaded into the wells of a 96-well microtiter plate. The biosensor strains were added into each well and the response of the biosensors after 4 h of incubation was analyzed with a SPECTRAmax^®^ GEMINI XS Microplate Spectrofluorometer (Molecular Devices, Sunnyvale, CA, United States). Experiments were performed in triplicates.

### Identification of AHL Degradation Metabolites

The activity of AHL-acylase was demonstrated with high-performance liquid chromatography (HPLC) analysis of the reaction mixtures containing chemically derivatized HSL rings using DANSYL chloride (5-dimethylamino-1-naphthalene sulfonyl chloride), as previously described (Lin et al., 2003). In brief, full-grown cultures of each isolate were mixed with 3 mM C_10_-HSL and incubated at 30°C for 3 h. The digestion mixtures were extracted thrice with equal volumes of ethyl acetate, and the extracted organic phases were evaporated to dryness. The samples were re-dissolved in 200 μL of methanol, and the resulting 100 μL solutions were reacted with an equal volume of DANSYL chloride (Tokyo Chemical Industry Co., Ltd., Tokyo, Japan, 2.5 mg/mL in acetone) at 40°C for 4 h. After evaporation to dryness, 50 μL of 0.2 M HCl was added to the sample to hydrolyze any excess DANSYL chloride. For HPLC analysis, the samples were introduced onto a Develosil ODS-UG-3 column (4.6 × 150 mm, Nomura Chemicals, Aichi, Japan). Fractions were isocratically eluted with 50:50 methanol–water (v/v) at a flow rate of 0.5 mL/min (Shimadzu SPD-6AV UV-VIS spectrophotometric detector, Shimadzu C-R6A chromatopac and Shimadzu SCL-6B system controller). A control experiment was performed with phosphate-buffered saline (PBS) instead of the strain solution. Quorum-quenching bacterium *Acidovorax* sp. strain MR-S7 known to possess AHL-acylase activity (Kusada et al., 2017) and 2 mM HSL standard (Sigma) were used as positive controls.

### Bioassay for AHL Production Activities

We performed the AHL production assay using GFP-based biosensor strain. In brief, the overnight culture fluids of isolates were extracted with equal volumes of ethyl acetate. The resulting liquid extractions were dispensed into the wells of a 96-well microtiter plate (Becton Dickinson, Franklin Lakes, NJ, United States) and treated with 50 μL of a fivefold-diluted overnight culture of the biosensor strain. The plate was statically incubated at 30°C for 4 h to induce detectable GFP expression from the reporter cell. *E. coli* strain (non-AHL producer) and C_10_-HSL solution were used as the negative and positive control, respectively. Experiments were performed in triplicate for each strain.

### Antibiotic Susceptibility Assay

Minimum inhibitory concentrations (MICs) of AHL-degrading bacteria were determined using microtiter plate dilution assays in R2A broth with about 1 × 10^5^ cells/well, as previously described (Yajko et al., 1987; Riesenfeld et al., 2004). MICs were determined after 1, 2, and 3 days of incubation at 30°C in the dark. MICs were read using a Multiskan^®^ Spectrum microplate spectrophotometer (Thermo Labsystems, Vantaa, Finland) and was defined as the lowest concentration of an antimicrobial agent at which the organism showed no visible growth (Yajko et al., 1987). *E. coli* strain EPI300^TM^ (Epicentre, Madison, WI, United States) was used as a negative control. The criteria used for the interpretation of antimicrobial susceptibility were based upon the achievable levels of antimicrobial agents. The tested antibiotics were PENG, AMP, AMO, carbenicillin (CAR), piperacillin (PIP), CEFL, and CEFD. All these antibiotics are in common use, and the environmental samples used in the present study were polluted with wastewater from PENG, AMP, AMO, CEFL, and CEFD production. The antibiotics were tested at concentrations of 8, 16, 32, 64, 125, 250, and 500 μg/mL.

### Chemicals

*N*-Hexanoyl-L-homoserine lactone (C_6_-HSL), *N*-octanoyl-L-homoserine lactone (C_8_-HSL), *N*-decanoyl-L-homoserine lactone (C_10_-HSL), *N*-dodecanoyl-L-homoserine lactone (C_12_-HSL), *N*-(3-oxo-hexanoyl)-L-homoserine lactone (3-oxo-C_6_-HSL), *N*-(3-oxo-octanoyl)-L-homoserine lactone (3-oxo-C_8_-HSL), and HSL standards were purchased from Sigma. *N*-(3-oxo-decanoyl)-L-homoserine lactone (3-oxo-C_10_-HSL), *N*-(3-oxo-dodecanoyl)-L-homoserine lactone (3-oxo-C_12_-HSL), and *N*-(3-oxo-tetradecanoyl)-L-homoserine lactone (3-oxo-C_14_-HSL) were obtained from the Nottingham University in England.

### Nucleotide Sequence Accession Numbers

The nucleotide sequences reported in this study were deposited in the GenBank database with accession numbers from AB646301 to AB646320.

## Results and Discussion

### Isolation of Antibiotic-Resistant Bacteria From Penicillin-Contaminated Environmental Samples

To isolate phylogenetically diverse PENG-resistant bacteria from the environmental samples polluted with wastewater from PENG production, we used gellan gum-solidified media supplemented with antibiotics (8, 16, 32, 64, 125, 250, and 500 μg/mL), as gellan was shown to be effective for cultivating phylogenetically novel and diverse bacteria (Tamaki et al., 2005, 2009). Ten and nine PENG-resistant individual colonies with characteristic morphologies and colors were isolated from the activated sludge and river sediment samples, respectively. The results of the 16S rRNA gene sequencing showed that these PENG-resistant isolates belong to at least 12 different genera across *Alphaproteobacteria, Betaproteobacteria, Gammaproteobacteria, Actinobacteria*, and *Bacteroidetes.* Of these, three isolates (S5, M9, and S15) showed low 16S rRNA gene sequence similarities (<97%) to any known bacterial species (Table 1).

**Table 1:** Phylogenetic affiliations of microbes grown on PENG amended medium on the basis of 16S rRNA gene sequences by using the BLAST program in the GenBank database.

Taxonomic group and strains ^a^	Closest species	Accession no.	Similarly (%)	Length (bp)
**Alpha-proteobacteria**		
S1	*Sphingomonas* sp. CC-MHH0539	KU248160	99	1240
S3	*Ochrobactrum pseudintermedium* ADV43	DQ365923	99	566
S20	*Bosea* sp. MF18	EF219051	99	556
**Beta-proteobacteria**		
S2	*Diaphorobacter* sp. GS-1	FJ158841	99	1489
S4	*Dechloromonas* sp. JDS6	AY084087	99	605
S5	*Dechloromonas* *agitata* A10	MG757543	92	568
S12	*Acidovorax temperans* Ls 4-1	KX622787	99	669
M8	*Dechloromonas* sp. Iso12-19	AB795533	99	611
M9	*Dechloromonas* sp. IsoEc.27	AB795517	96	569
M2	*Acidovorax* sp. Iso-33	KC768746	99	1488
M6	*Acidovorax* sp. Iso-33	KC768746	99	1465
M14	*Hydrogenophaga* *defluvii*	AM942546	98	577
**Gamma-proteobacteria**		
S15	*Pseudoxanthomonas kaohsiungensis* J36	AY650027	95	615
S17	*Stenotrophomonas* sp. LMG 19833	AJ300772	99	1476
M18	*Morganella morganii* FUA1245	HQ169126	98	665
M20	*Enterobacter cloacae* M380	HQ651838	97	611
M21	*Stenotrophomonas* sp. ICB209	FJ748674	98	660
**Bacteroidetes**		
S16	*Elizabethkingia* sp. ds13-11	HQ436416	99	598
**Actinobacteria**		
M1	*Mycobacterium chelonae* B14	JX010972	99	1481

*^*a*^ S: Activated sludge; M: River sediment. As for AHL-degrading bacteria, the almost full 16S rRNA gene sequences (≈1,500 bp) were determined*.

### Exploration of Novel AHL-Degrading Bacteria Among the PENG-Resistant Isolates

The PENG-resistant isolates were tested for QQ activity using GFP-based AHL biosensors. Based on the preliminary screening using biosensors toward 3-oxo-C_6_-HSL and 3-oxo-C_12_-HSL, six of the PENG-resistant strains exhibited AHL-degrading activity, as observed with biosensors toward 3-oxo-C_12_-HSL after a 15 h incubation period (Supplementary Table 1). Thereafter, we selected the six resistant strains harboring high AHL-degrading ability (more than 50% degradation of the initial AHL) from the preliminary screening to further investigate QQ behaviors. Six isolates showed high capabilities of inactivating a broad range of AHLs, including AHLs with 3-oxo substituents. In particular, these strains exhibited higher QQ activities toward AHLs with long acyl chains than those with short acyl chains (Table 2).

**Table 2:** AHL-degrading behaviors of PENG resistant isolates^a^.

Strain	AHL congeners								
	C_6_	C_8_	C_10_	C_12_	OC_6_	OC_8_	OC_10_	OC_12_	OC_14_
S1	-	+++	+++	++	-	-	+	+	+
S2	-	+++	+++	++	-	++	+++	+++	+
S17	-	+++	+++	++	-	-	+++	+++	+
M1	-	+++	+++	++	-	-	+++	+++	+
M2	-	-	++	++	-	-	+++	+++	+
M6	+	-	+++	++	-	-	+++	+++	+

*^*a*^Biosensors for AHLs: *E. coli* JB525-MT102 (pJBA132) was used as s sensor strain for detecting C_*6*_-HSL, C_*8*_-HSL, C_*10*_-HSL, C_*12*_-HSL, 3-oxo-C_*6*_-HSL (OC_*6*_), 3-oxo-C_*8*_-HSL (OC_*8*_), 3-oxo-C_*10*_-HSL (OC_*10*_), 3-oxo-C_*12*_-HSL (OC_*12*_), and *P. putida* F117 (pKR-C12) was used for detecting 3-oxo-C_*14*_-HSL (OC_*14*_). The amount of remaining AHL was measured using bioassay after cultivation in a medium containing 20 μM AHL and biosensors for 4 h. The number of plus symbol relates to the amount of AHL degraded: +, 20–50% degradation of initial AHL; ++, 50–80%; +++, 80–100%*.

**FIGURE 2:**
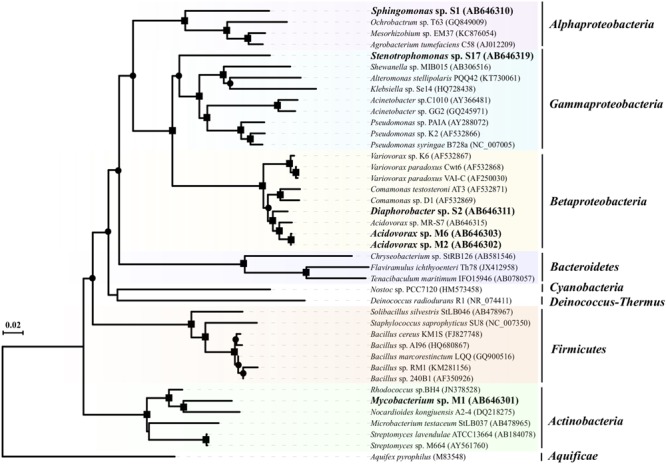
Phylogenetic affiliations of the AHL-degrading isolates obtained in this study and the previously known AHL degraders based on their almost full length 16S rRNA gene sequences. The phylogenetic tree was constructed by neighbor-joining (NJ) method with Kimura’s correction. The 16S rRNA gene sequence of *Aquifex pyrophilus* (M83548) was used as an outgroup. Bootstrap values of >50% and >80% estimated using neighbor-joining and maximum-likelihood methods (1,000 replications) are shown by circle and square at branching points, respectively. The six new AHL-degrading strains isolated in this study are shown in name with boldface.

Phylogenetic analysis based on almost full-length 16S rRNA gene sequences showed that the six QQ isolates were associated with three Gram-negative taxa, *Alphaproteobacteria* (*Sphingomonas* sp. S1), *Betaproteobacteria* (*Acidovorax* sp. M2, *Acidovorax* sp. M6 and *Diaphorobacter* sp. S2), and *Gammaproteobacteria* (*Stenotrophomonas* sp. S17) and one Gram-positive taxon, *Actinobacteria* (*Mycobacterium* sp. M1) (Figure 2). Although phylogenetically diverse QQ bacteria have been isolated so far, a Gram-positive bacterium within the genus *Mycobacterium* has never been shown to exhibit QQ activity.

To determine whether the six AHL degraders produce their own AHL isomers, a GFP-based biosensor strain was used (see method). The ethyl acetate extracts of six isolates as well as the negative control (non-AHL producer *E. coli*) showed low values of GFP fluorescence (Supplementary Figure 1). This result indicates that these six isolates lack the ability to produce biosensor-detectable AHL-like compounds. Although these six isolates were unable to produce AHLs, they might have the ability to sense exogenous AHLs produced by other bacteria, and the resulting QS system would enhance biofilm formation and antibiotic resistance. Indeed, our previous study demonstrated that a wide variety of exogenous AHLs induced biofilm formation of non-AHL producer, *Acidovorax* sp. strain MR-S7, and the MICs of AHL-supplemented (biofilm-forming) strain MR-S7 showed 5–10 folds higher resistance to various antibiotics (Kusada et al., 2014). Most human infections are localized within biofilms, where QS signaling is much more efficient due to localization of AHLs. The genetic information of these isolates (e.g., *luxR* gene encoding the AHL-responsive transcriptional regulator) and biochemical experiments provided in the future study would further verify the effect of QS on biofilm formation and antibiotic resistance.

### Identification of AHL Degradation Products

**FIGURE 3:**
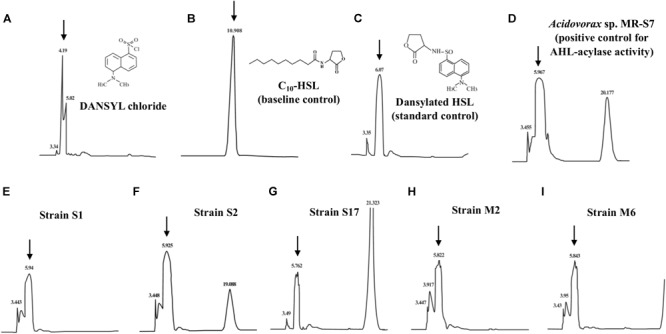
Metabolite analysis of C_10_-HSL degradation products by cell extract of the AHL degrading isolates. HPLC profiles of unreacted DANSYL chloride solution **(A)**, C_10_-HSL **(B)**, dansylated HSL standard **(C)**, reaction product of C_10_-HSL after incubation with cell extract of *Acidovorax* sp. MR-S7 **(D)**, strain S1 **(E)**, strain S2 **(F)**, strain S17 **(G)**, strain M2 **(H)**, and strain M6 **(I)**. Note that a dansylated digestion product and a dansylated HSL standard both eluted with retention time at 5.8–6.0 min.

To demonstrate the AHL degradation mechanism of these six isolates, HPLC analysis was performed to detect the presence of HSL ring generated by AHL-acylase activities. To determine whether HSL was released as an AHL degradation product, samples of the reaction mixture were treated with DANSYL chloride and analyzed by HPLC. The LC retention time of DANSYL chloride was 4.19 min, whereas that of the dansylated digestion mixtures from isolates (S1, S2, S17, M2, and M6) was around 6.0 min, identical to the retention time of the standard control of dansylated HSL and the dansylated digestion mixture from *Acidovorax* sp. MR-S7 known to degrade AHLs by AHL-acylase activity (Figure 3 and Supplementary Figure 2). These results indicate that the five isolates were capable of degrading C_10_-HSL with their AHL-acylase activities. Note that the remaining one isolate, strain M1, was able to degrade a wide range of AHLs but did not show AHL-acylase activity in the HPLC assay. This likely suggests that the strain M1 may possess the distinct degradation mechanism such as AHL-lactonase activity, though further investigation would be needed to reveal their QQ activities using purified enzymes.

β-Lactam antibiotics and AHLs are structurally similar as both compounds have ring structures and acyl side chains. In recent years, AHL-acylase enzymes were found to be also phylogenetically and structurally similar to β-lactam antibiotic resistance enzymes, β-lactam acylases. Besides, our group and other two research groups reported that AHL-acylases (MacQ, AhlM, and KcPGA) could function as β-lactam acylase, and indeed degrade PENG via hydrolysis of the amide bond (Park et al., 2005; Mukherji et al., 2014; Kusada et al., 2017). Furthermore, we recently solved the X-ray crystal structure of MacQ, and found that the degradation products of C_10_-HSL and PENG by MacQ were commonly accommodated in the same hydrophobic active-site pocket, indicating that both compounds were hydrolyzed by MacQ in the same degradation mechanism (Yasutake et al., 2017). Perhaps, some AHL-acylases (β-lactam acylases) may have broad substrate specificity that appears to be structurally indistinguishable from both AHL-signals and β-lactam antibiotics. Further genetic and biochemical analyses of other AHL-acylases (β-lactam acylases) will be required to more fully assess the mechanism and relationships between QQ and antibiotic resistance.

### β-Lactam Antibiotic Resistance Assay

We measured the MICs of seven different β-lactam antibiotics, including PENG, AMP, AMO, CAR, PIP, CEFL, and CEFD, toward the six AHL-degrading isolates (Table 3). In comparison with the control strain *E. coli* EPI300^TM^, all of the AHL-degrading isolates displayed a high resistance profile to almost all β-lactam antibiotics examined. In addition, the comparison of the antibiotic resistance activities of the six isolates with those of the previously identified multiple β-lactam antibiotic resistant pathogens revealed that our isolates displayed comparable or even greater values of MICs to all of the seven β-lactam antibiotics tested (John and McNeill, 1980; Colom et al., 1995; Stapleton et al., 1995; Lauretti et al., 1999; Afzal-Shah et al., 2001; Dubois et al., 2002). In particular, strains S17 and M2 displayed high abilities to resist broad types of β-lactam antibiotics. The MICs for these strains were over 500 μg/mL of PENG, AMP, AMO, CAR, PIP, CEFL, and CEFD. These values were at least 31.3- to 62.5-fold higher than the values observed for the control strain *E. coli* EPI300^TM^ (<8–16 μg/mL) (Table 3). Furthermore, the MICs of AMP, PIP, and CAR were over 30-fold higher for the six AHL-degrading isolates than for the control strain *E. coli* EPI300^TM^.

**Table 3:** Sensitivity of isolates to β-lactam antibiotics^a^.

Strain	MIC (μg/ml)						
	PENG	AMP	AMO	CAR	PIP	CEFL	CEFD
S1	250	>500	250	>500	500	500	250
S2	>500	>500	500	>500	250	500	500
S17	>500	>500	>500	>500	>500	>500	>500
M1	125	>500	64	>500	>500	250	125
M2	>500	>500	>500	>500	>500	>500	>500
M6	500	>500	500	500	250	32	8
*E. coli* EPI300	32	16	8	16	8	16	16

*^*a*^ Penicillin G (PENG); ampicillin (AMP); amoxicillin (AMO); carbenicillin (CAR); piperacillin (PIP); cefalexin (CEFL); cefadroxil (CEFD). The MIC (minimum inhibitory concentration) was defined as the lowest concentration of antibiotics at which the organism showed no visible growth*.

In total, six AHL-degrading isolates exhibited high antibiotic resistance and broad substrate specificities toward multiple β-lactams as well as AHL isomers. The knowledge relevant to such organisms possessing both multiple β-lactam antibiotic resistance and AHL degradation activities has been limited. Two multiple β-lactam antibiotic resistant bacteria, *P. aeruginosa* PAO1 and *Acidovorax* sp. MR-S7, have been reported to be able to degrade AHLs. In comparison to *P. aeruginosa* PAO1, our six isolates showed much higher resistance to β-lactams tested (MICs of AMP, CAR, and PIP toward strain PAO1 were 256, 64, and 4 μg/mL, respectively). Given that these six isolates degrading multiple β-lactams and AHLs were found across two phyla and four classes, our findings implicate a possibility that this versatile phenotypic bi-functionality may be more broadly present in bacteria dwelling in natural ecosystems and provide new insight into the diversity of organisms that simultaneously exhibit both multiple β-lactam antibiotic resistance and QQ activities. To further clarify the functional and evolutionary correlation between β-lactam antibiotic resistance and QQ activities, studies are warranted to identify the genetic elements responsible for conferring multiple β-lactam antibiotic resistance and QQ activities.

## Conclusion

In this study, we successfully isolated six novel QQ bacteria from β-lactam antibiotic-resistant isolates obtained from PENG-polluted environmental samples and characterized their AHL- and β-lactam antibiotic-degrading properties. This study expands the diversity of organisms that possess two different and important physiological functions, QQ activity and β-lactam antibiotic resistance. In addition, our study provides a novel screening strategy for the identification of AHL-degrading and β-lactam antibiotic-resistant bacteria previously unidentified. Taken together with previous studies, our findings provide additional evidence that AHL-degrading bacteria may be the potential multiple β-lactam antibiotic resistant candidates that have long been overlooked.

## Author Contributions

YZ, HT, NK, and YK conceived the study and designed experiments. HK, YZ, NK, and HT performed experiments and analyzed the data. HK, HT, NK, and YK wrote the manuscript. All authors contributed to the discussion of the results obtained in this study, and reviewed and edited the manuscript.

## Conflict of Interest Statement

The authors declare that the research was conducted in the absence of any commercial or financial relationships that could be construed as a potential conflict of interest.

## Footnotes

1 www.ncbi.nlm.nih.gov/BLAST
