# Supplementary material for: Novel N-Acyl Homoserine Lactone-Degrading Bacteria Isolated From Penicillin-Contaminated Environments and Their Quorum-Quenching Activities
Source: Front Microbiol. 2019 Mar 14;10:455. doi: 10.3389/fmicb.2019.00455 (PMC6426785; doi:10.3389/fmicb.2019.00455)
Supplement: Supplementary file 1 [file Data_Sheet_1.docx]

Supplementary Material

**Novel *N*-acylhomoserine lactone-degrading bacteria isolated from penicillin-contaminated environments and their quorum-quenching activities**

Hiroyuki Kusada, Yu Zhang, Hideyuki Tamaki*, Nobutada Kimura and Yoichi Kamagata*

*** Correspondence:** Hideyuki Tamaki ([tamaki-hideyuki@aist.go.jp)](mailto:tamaki-hideyuki@aist.go.jp)); Yoichi Kamagata ([y.kamagata@aist.go.jp](mailto:y.kamagata@aist.go.jp))

# Supplementary Figure


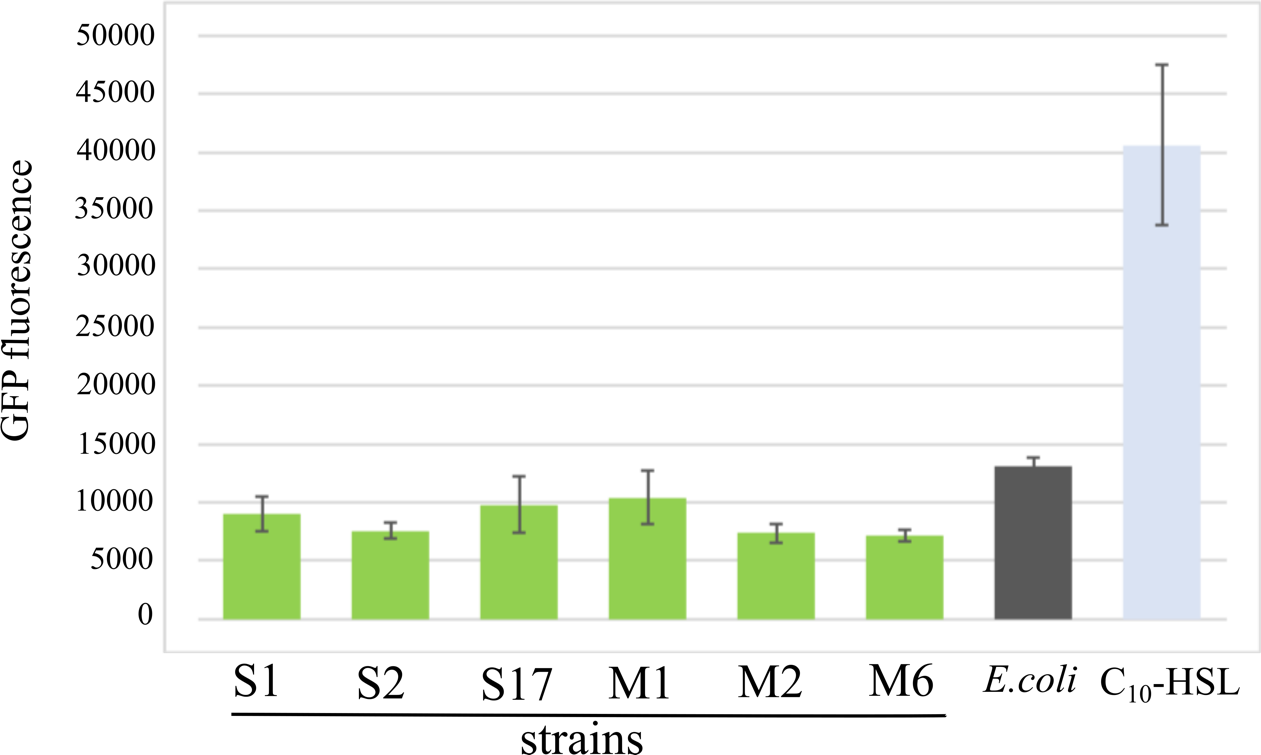


**Supplementary Figure 1.**  AHL-production assay using GFP-based biosensor strain. The y-axis indicates GFP fluorescence intensity. *E. coli* strain (non-AHL producer) and C_10_-HSL solution were used as negative and positive control, respectively. Values are expressed as means for three technical replicates. Error bars indicate standard deviation (SD).


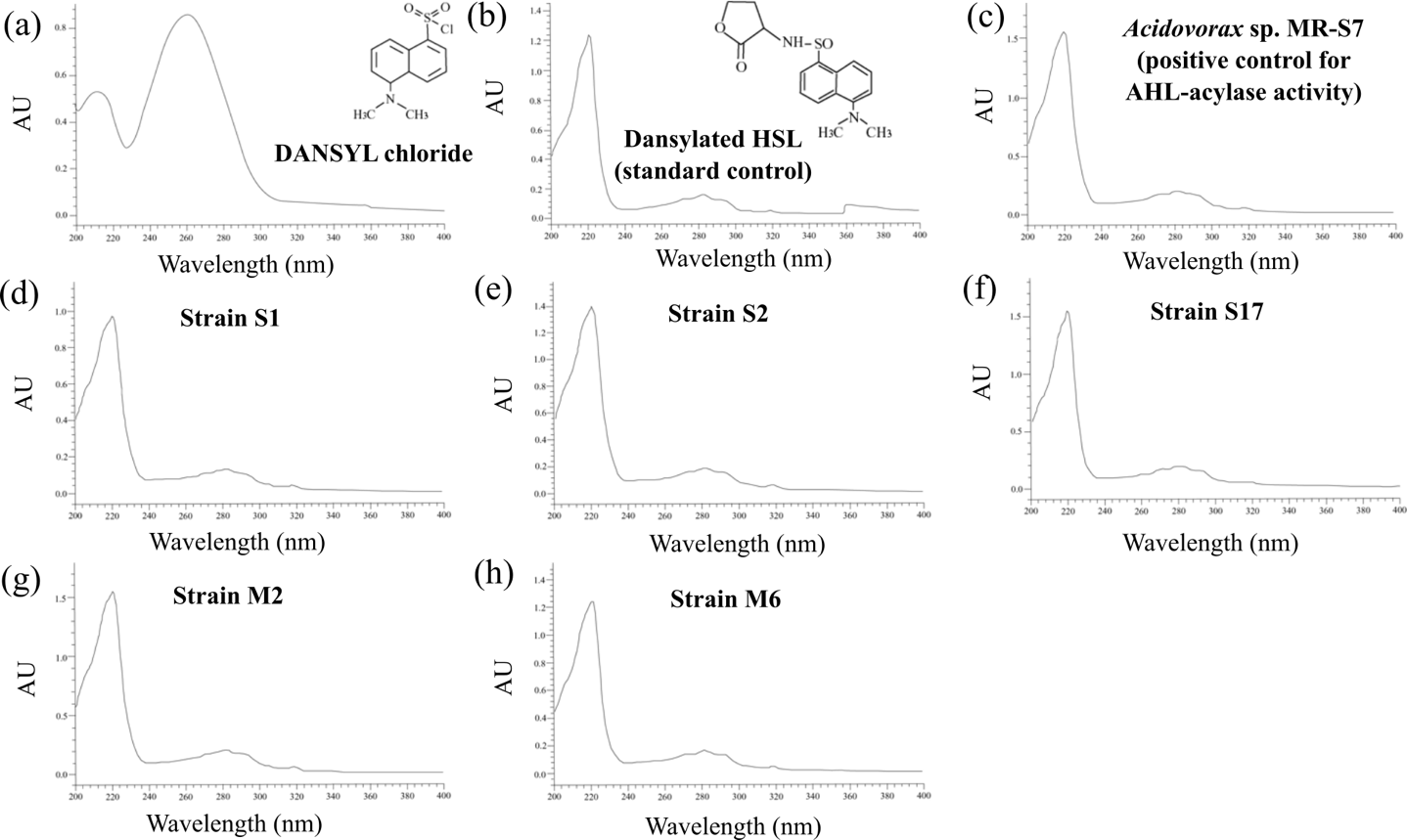


**Supplementary Figure 2.**  UV absorbance profiles of DANSYL chloride (a), dansylated HSL standard (b), and dansylated C_10_-HSL degraded by cell extract of AHL-degrading strain MR-S7 known to degrade AHLs by AHL-acylase (c), strain S1 (d), strain S2 (e), strain S17 (f), strain M2 (g), and strain M6 (h). The absorbance maxima for DANSYL chloride and its dansylated HSL derivative are 265 and 220 nm, respectively.

**2. Supplementary Table**

Supplementary Table 1. Initial screening for AHL-degrading activities of PENG resistant isolates^a^

| Strains | AHL congeners | |
| --- | --- | --- |
|  | OC_6_-HSL | OC_12_-HSL |
| S1 | - | + |
| S2 | - | +++ |
| S3 | - | - |
| S4 | - | - |
| S5 | - | - |
| S12 | - | - |
| S15 | - | - |
| S16 | - | - |
| S17 | - | +++ |
| S20 | - | - |
| M1 | - | +++ |
| M2 | - | +++ |
| M6 | - | +++ |
| M8 | - | - |
| M9 | - | - |
| M14 | - | - |
| M18 | - | - |
| M20 | - | - |
| M21 | - | - |

^a^ Biosensors for AHLs: *E.coli* JB525-MT102 (pJBA132) was used as s sensor strain for detecting 3-oxo-C_6_-HSL (OC_6_) and 3-oxo-C_12_-HSL (OC_12_). The amount of remaining AHL was measured using bioassay after cultivation in a medium containing 20 μM AHL and biosensors for 4h. The number of plus symbol relates to the amount of AHL degraded: +, 20-50% degradation of initial AHL; ++, 50-80%; +++, 80-100%.
